# *JAG1* Mutation Spectrum and Origin in Chinese Children with Clinical Features of Alagille Syndrome

**DOI:** 10.1371/journal.pone.0130355

**Published:** 2015-06-15

**Authors:** Liting Li, Jibin Dong, Xiaohong Wang, Hongmei Guo, Huijun Wang, Jing Zhao, Yiling Qiu, Kuerbanjiang Abuduxikuer, Jianshe Wang

**Affiliations:** 1 Center for Pediatric Liver Diseases, Children’s Hospital of Fudan University, Shanghai, China; 2 School of Pharmacy, Fudan University, Shanghai, China; 3 Department of Gastroenterology, Nanjing Children's Hospital Affiliated to Nanjing Medical University, Nanjing, Jiangsu, China; 4 The Molecular Genetic Diagnosis Center, Shanghai Key Lab of Birth Defect, Translational Medicine Research Center of Children Development and Disease, Pediatrics Research Institute, Children’s Hospital of Fudan University, Shanghai, China; 5 Department of Pediatrics, Jinshan Hospital of Fudan University, Shanghai, China; Ohio State University Medical Center, UNITED STATES

## Abstract

Alagille syndrome is an autosomal dominant disorder that results from defects in the Notch signaling pathway, which is most frequently due to *JAG1* mutations. This study investigated the rate, spectrum, and origin of *JAG1* mutations in 91 Chinese children presenting with at least two clinical features of Alagille syndrome (cholestasis, heart murmur, skeletal abnormalities, ocular abnormalities, characteristic facial features, and renal abnormalities). Direct sequencing and/or multiplex-ligation-dependent probe amplification were performed in these patients, and segregation analysis was performed using samples available from the parents. *JAG1* disease-causing mutations were detected in 70/91 (76.9%) patients, including 29/70 (41.4%) small deletions, 6/70 (8.6%) small insertions, 16/70 (22.9%) nonsense mutations, 8/70 (11.4%) splice-site mutations, 6/70 (9.4%) missense mutations, and 5/70 (7.1%) gross deletions. Of the mutations detected, 45/62 (72.6%) were novel, and almost all were unique, with the exception of c.439C>T, c.439+1G>A, c.703C>T, c.1382_1383delAC, c.2698C>T, and c.2990C>A, which were detected in two cases each; three cases exhibited entire gene deletions. A majority (69.2%) of the point and frameshift mutations could be detected by the sequencing of eleven exons (exons 3, 5, 6, 11, 14, 16, 18, 21, and 23–25). The mutation detection rate was 50.0% (10/20) in atypical cases that only presented with two or three clinical features of Alagille syndrome. Segregation analysis revealed that 81.1% (30/37) of these mutations were *de novo*. In conclusion, *JAG1* mutations are present in the majority of Chinese pediatric patients with clinical features of Alagille syndrome, and the mutations concentrate on different exons from other reports. Genetic study is important for the diagnosis of atypical Alagille syndrome in Chinese patients.

## Introduction

Alagille syndrome (ALGS; OMIM 118450) is an autosomal dominant disorder that results from defects in the Notch signaling pathway, typically via mutations in the gene encoding a ligand for Notch receptors, *JAGGED1* (*JAG1*). ALGS is associated with a wide variety of clinical features and manifestations, including abnormalities of the liver, heart, skeleton, eyes, kidneys, and facial features [1]. It is one of the most common causes of pediatric chronic liver disease and occurs with a minimal estimated frequency of 1 in 70,000–100,000 newborn infants [2]. The classical criteria for ALGS diagnosis include bile duct paucity on liver biopsy in association with three of the following: cholestasis, congenital heart disease, vertebral abnormalities, characteristic facial features, and posterior embryotoxon [3]. However, the advent of molecular diagnostic testing has led to a revision of diagnostic criteria for ALGS [4].

*JAG1* mutations and/or ALGS clinical features have been reported in various populations, such as American, European, Australian, and Japanese [5–10]. Nearly 500 *JAG1* mutations have been identified (HGMD Professional 2015.1). Approximately 94% of patients with a clinically confirmed diagnosis of ALGS carry *JAG1* mutations, of which 60–70% are *de novo* [11–13]. We previously reported a case series of Chinese ALGS patients with a *de novo* mutation frequency of 100% (5/5), including an atypical disease case [14]. To further characterize *JAG1* mutations and their origins in Chinese patients with ALGS, we performed a genetic study on a cohort of sporadic patients with at least two of the six major clinical features (chronic cholestasis, cardiac murmur, skeletal abnormalities, ocular abnormalities, a characteristic face, and renal abnormalities).

## Materials and Methods

### Subjects

This study included 91 patients (37 female and 54 male, including 17 reported previously [14]) from 89 unrelated families referred to the pediatric liver disease clinic of Children’s Hospital of Fudan University between January 2010 and December 2014; cases 30 and 70 were twins, and cases 59 and 69 were brothers.

### Diagnostic criteria for ALGS and evaluations of clinical features

All patients had at least two clinical features of ALGS (Table 1). The diagnosis of ALGS was based on the presence of bile duct paucity and at least three major clinical features, including chronic cholestasis, cardiac murmur, skeletal abnormalities, ocular abnormalities, and a characteristic face, or at least four of six major clinical features (chronic cholestasis, cardiac murmur, skeletal abnormalities, ocular abnormalities, a characteristic face and renal abnormalities) in the absence of paucity of bile ducts [4].

**Table 1:** Pathologic and clinical Alagille syndrome features of 91 patients.

Patient No.	Interlobular bile duct paucity	Cholestasis	Cardiac murmur	Skeletal abnormalities	Characteristic face	Posterior embryotoxon	Kidney abnormalities	Total clinical features, *n*
1*	+	+	+	+	+	+	+	6
2*	NA	+	+	+	+	+	+	6
3*	+	+	+	+	+	+	-	5
4*	+	+	+	+	+	+	-	5
5*	+	+	+	-	+	+	+	5
6*	+	+	+	+	+	+	-	5
7*	+	+	+	+	+	+	-	5
8*	-	+	+	+	+	+	-	5
9*	+	+	+	+	+	+	-	5
10*	NA	+	+	+	+	+	-	5
11*	NA	+	+	+	+	+	-	5
12*	NA	+	+	+	+	-	+	5
13*	NA	+	+	+	+	+	-	5
14*	NA	+	+	+	+	+	-	5
15*	NA	+	+	+	+	-	+	5
16*	NA	+	+	+	+	+	-	5
17*	NA	+	+	+	+	+	-	5
18*	NA	+	+	+	+	-	+	5
19*	+	+	+	+	+	NA	+	5
20*	NA	+	+	+	+	+	NA	5
21*	+	+	+	+	+	+	NA	5
22*	-	+	+	+	+	+	NA	5
23*	NA	+	+	+	+	+	NA	5
24*	+	+	+	-	+	+	-	4
25*	+	+	-	+	+	-	+	4
26*	+	+	+	+	+	-	-	4
27*	-	+	+	+	+	-	-	4
28*	-	+	+	+	+	-	-	4
29*	NA	+	+	+	+	-	-	4
30*	NA	+	+	+	+	-	-	4
31*	NA	+	+	-	+	-	+	4
32*	NA	+	+	+	+	-	-	4
33*	NA	+	+	+	-	+	-	4
34*	NA	+	+	-	+	+	-	4
35*	NA	+	+	+	+	-	-	4
36*	+	+	+	+	+	-	NA	4
37*	-	+	+	-	+	+	NA	4
38*	NA	+	+	+	+	-	NA	4
39*	NA	+	+	+	+	-	NA	4
40*	NA	+	+	+	+	NA	-	4
41*	NA	+	+	+	+	NA	-	4
42*	NA	+	+	+	+	NA	-	4
43*	+	+	+	+	+	NA	-	4
44*	NA	+	+	+	+	-	NA	4
45*	-	+	+	+	+	-	NA	4
46*	+	+	+	+	+	NA	NA	4
47*	+	+	+	+	-	-	-	3
48*	+	+	+	+	-	NA	NA	3
49	NA	+	+	+	+	+	-	5
50	-	+	+	+	+	+	-	5
*51*	+	+	+	+	+	+	-	5
*52*	NA	+	+	+	+	-	+	5
53	+	+	-	+	+	-	+	4
54	NA	+	+	-	+	+	-	4
55	+	+	+	-	+	-	-	3
56*	NA	+	+	+	-	-	-	3
57*	NA	+	+	+	-	-	-	3
58*	NA	+	+	+	-	-	-	3
59*	NA	+	+	-	-	+	-	3
60*	NA	+	+	-	+	-	-	3
61*	NA	+	-	+	+	-	-	3
62*	NA	+	-	+	+	-	-	3
63	NA	+	+	+	-	-	-	3
64	-	+	+	-	+	-	-	3
65	NA	+	+	-	-	+	-	3
66*	NA	+	+	-	+	-	NA	3
67*	NA	+	-	+	+	-	NA	3
68*	NA	+	+	+	-	-	NA	3
69*	NA	+	+	-	+	-	NA	3
70*	NA	+	+	-	+	NA	-	3
71*	NA	+	+	-	+	NA	-	3
72*	NA	+	-	+	+	NA	-	3
73*	NA	+	+	-	+	NA	-	3
74*	-	+	+	+	-	NA	NA	3
75*	NA	+	+	+	-	NA	NA	3
76*	NA	+	+	+	NA	NA	NA	3
77	NA	+	+	+	-	NA	NA	3
78*	-	+	+	-	-	-	-	2
79	NA	+	-	-	+	-	-	2
80	+	+	+	-	-	-	-	2
81*	NA	+	-	+	-	-	NA	2
82*	NA	+	-	-	+	NA	-	2
83	NA	+	-	-	-	NA	+	2
84	NA	+	-	+	-	-	NA	2
85	NA	+	+	-	-	NA	-	2
86	NA	+	+	-	-	NA	-	2
87	NA	+	-	NA	+	-	-	2
88*	NA	+	-	+	-	NA	NA	2
89	NA	+	+	-	-	NA	NA	2
90	NA	-	+	NA	-	+	NA	2
91	NA	+	+	NA	-	NA	-	2
Total	31	91	91	88	90	69	66	

NA: not available.

*JAG1 mutation detected; cases previously reported are in italic font.

Cases 1–55 met the clinical diagnostic criteria for Alagille syndrome; cases 56–91 were considered as clinically suspected cases.

Ninety of the 91 patients were initially referred to our center primarily for cholestasis, including 83 cases with jaundice and 7 cases presenting with pruritus and elevated serum transaminase with high γ-glutamyl transpeptidase. One case initially presented with hepatomegaly. The majority of physical examinations were performed by one author (JSW). The presence of a heart murmur or ALGS facial features prompted additional examinations, including echocardiography, abdominal ultrasound, radiography of the spine, and ophthalmologic examination. Results of these examinations, along with clinical features and liver function test results were retrospectively obtained from medical records. Liver biopsy was performed on 31 patients. Histology of all patients was assessed by the same experienced liver pathologist.

### Mutation detection

With the approval of the ethics committee of Children’s Hospital of Fudan University and written informed consent from parents, ~1 mL of peripheral blood was obtained from each participant and his/her parents (if available). Genomic DNA from peripheral blood lymphocytes was extracted using commercial extraction kits. All 26 coding exons of *JAG1* (RefSeq NM_000214.2) including at least 100 bp of adjacent intronic sequence were amplified by PCR (primer sequences available on request), and detected by laser-induced fluorescence on an ABI Prism 3130 or 3500 Genetic Analyzer (Applied Biosystems of Thermo Fisher Scientific, Waltham, MA, USA). Sequence analysis was performed using BIOEDIT software (North Carolina State University, Raleigh, NC, USA) and doubly checked by two investigators. All sequences were compared using BLAST against genomic sequences from the National Center for Biotechnology Information. If no mutation was detected by sequencing, multiplex-ligation-dependent probe amplification (MLPA) dosage analysis was carried out to look for partial or whole gene deletions. MLPA analysis was performed according to the manufacturer’s instructions using the P184 MLPA kit available from MRC-Holland (Amsterdam, Netherlands).

The pathogenicity of missense variants was analyzed using Mutation Taster (http://www.mutationtaster.org) and Polyphen-2 (http://genetics.bwh.harvard.edu/pph-2). Additional factors that were considered include: (a) absence in the general population; (b) novel appearance and disease phenotype from the family pedigree; (c) absence of any other mutation in *JAG1* that could be responsible for the clinical phenotype; and (d) previous independent occurrence in an unrelated patient.

## Results

### Mutations and polymorphisms

Sequence analysis was successful for all cases, and an MLPA dosage result was obtained for 22 cases in which no mutation or only a missense variant was identified by sequencing and sufficient DNA was available. Sequencing and MLPA identified 62 different mutations in these patients. The mutations were unique among cases, with the exceptions of c.439C>T, c.439+1G>A, c.703C>T, c.1382_1383delAC, c.2698C>T, and c.2990C>A, which occurred in two cases, and entire gene deletions in three cases. Fifty-nine mutations were identified by sequencing, including frameshift (*n* = 34), nonsense (*n* = 12), splicing site (*n* = 7), and missense (*n* = 6) mutations. Of these mutations, 72.6% (45/62) were novel (Table 2).

**Table 2:** Summary of *JAG1* mutations identified in patients.

Patient No.	Sex	Mutation	Location	Domain	Origin
*1*	*Male*	***c*.*1868delG*, *p*.*G623EfsX118*, *het***	Exon 14	EGF	ND
*2*	*Female*	*c*.*439C>T*, *p*.*Q147X*, *het*	Exon 3	5´ of DSL	ND
*3*	*Male*	***c*.*866delG*, *p*.*G289AfsX121*, *het***	Exon 6	EGF	*De novo*
*4*	*Male*	***c*.*1323_1326delCTGG*, *p*.*M443VfsX4*, *het***	Exon 10	EGF	ND
*5*	*Female*	***c*.*1771_1775delGTGCG1insT*, *p*.*V591CfsX149*, *het***	Exon 14	EGF	ND
6	Female	**c.2628G>A, p.W876X, het**	Exon 22	CR	*De novo*
7	Female	**c.439+2dupT, het**	Intron 3		*De novo*
*8*	*Male*	***c*.*550C>T*, *p*.*R184C*, *het;*** *MLPA not done*	Exon 4	5´ of DSL	*De novo*
9	Male	c.2572+1G>T, het	Intron 21		ND
10	Female	**c.980_989delGGTATTCAGG, p.G327DfsX82, het**	Exon 7	EGF	ND
11	Male	c.1007delC, p.A336VfsX76, het	Exon 8	EGF	De novo
12	Male	**c.2230delC, p.R744EfsX76, het**	Exon 18	EGF	*De novo*
13	Male	**c.2502delC, p.C835VfsX35, het**	Exon 21	EGF	*De novo*
14	Female	c.703C>T, p.R235X, het	Exon 5	5´ of EGF	Paternal
15	Female	c.2473C>T, p.Q825X, het	Exon 21	EGF	Paternal
16	Female	c.2698C>T, p.R900X, het	Exon 23	CR	ND
17	Female	**c.3140C>A, p.S1047X, het**	Exon 25	5´ of TM	*De novo*
18	Male	entire gene deletion, het			ND
19	Female	**c.1148_1149delGT, p.C383FfsX11, het**	Exon 9	EGF	ND
*20*	*Male*	***c*.*3099_3100delCA*, *p*.*D1033EfsX5*, *het***	Exon 25	CR	*De novo*
*21*	*Male*	*c*.*2230C>T*, *p*.*R743X*, *het*	Exon 18	EGF	ND
22	Male	**c.1349-10_1353delTATTTTTTAGATATT, het**	Intron 10-Exon11		*De novo*
*23*	*Female*	*c*.*1156G>A*, *p*.*G386R*, *het; MLPA not done*	Exon 9	EGF	ND
24	Male	**c.410delA, p.E137GfsX24, het**	Exon 3	5´ of DSL	ND
*25*	*Male*	*c*.*693_694delAG*, *p*.*R231SfsX8*, *het*	Exon 4	5´ of DSL	ND
26	Male	**c.1468G>T, p.E490X, het**	Exon 12	EGF	*De novo*
*27*	*Male*	*c*.*439+1G>A*, *het*	Intron 3		*De novo*
28	Male	**Ex.1-5 deletion, het**			ND
29	Male	**c.755+2T>G, het**	Intron 5		Maternal
30	Female	**c.1382_1383delAC, p.D461GfsX8, het**	Exon 11	EGF	*De novo*
31	Male	**c.1842delC, p.C615VfsX128, het**	Exon 14	EGF	*De novo*
32	Male	**c.1859delG, p.G620AfsX123, het**	Exon 14	EGF	*De novo*
33	Male	**c.2909_2913delTGTCA, p.M970TfsX11, het**	Exon 23	CR	*De novo*
34	Female	**c.2070_2073dupCTGT, het**	Exon 16	EGF	ND
35	Female	**c.3088_3089insG, p.E1030GfsX4, het**	Exon 25	5´ of TM	ND
36	Male	c.439C>T, p.Q147X, het	Exon 3	5´ of DSL	*De novo*
*37*	*Female*	***c*.*766G>T*, *p*.*G256C*, *het;*** *MLPA not done*	Exon 6	EGF	*De novo*
38	Male	**c.2026T>G, p.C676G, het** and no mutation by MLPA	Exon 16	EGF	*De novo*
39	Male	**c.3008_3020insAGCCTTCCCCTTC, p.E1030GfsX4, het**	Exon 24	CR	Maternal
40	Female	c.2225_2226delTA, p.I742SfsX5, het	Exon 17	EGF	Maternal
41	Female	**c.702C>A, p.C234X, het**	Exon 5	5´ of EGF	*De novo*
42	Female	**c.238A>G, p.K80E, het** and no mutation by MLPA	Exon 2	5´ of DSL	*De novo*
43	Female	entire gene deletion, het			ND
44	Male	entire gene deletion, het			ND
45	Female	**Ex.2-26 deletion, het**			ND
*46*	*Female*	***c*.*2791_2792insA*, *p*.*T931NfsX19*, *het***	Exon 23	CR	ND
*47*	*Male*	***c*.*819delC*, *p*.*H273QfsX*, *het***	Exon 6	EGF	ND
48	Female	c.1899_1900delTG, p.C633X, het	Exon 15	EGF	*De novo*
49	Female	No mutation by sequencing and MLPA			
50	Female	No mutation by sequencing and MLPA			
*51*	*Male*	*No mutation by sequencing; MLPA not done*			
*52*	*Male*	*No mutation by sequencing; MLPA not done*			
53	Male	No mutation by sequencing and MLPA			
54	Female	No mutation by sequencing and MLPA			
55	Male	No mutation by sequencing; MLPA not done			
56	Male	c.1499delG, p.G500VfsX64, het	Exon 12	EGF	ND
57	Male	**c.2314delG, p.E772KfsX48, het**	Exon 18	EGF	ND
58	Male	**c.3244_3256delATCTGTTGCTTGG, het**	Exon 26	TM	*De novo*
59	Male	**c.2990C>A, p.S997X, het**	Exon 24	CR	Maternal
60	Male	c.3031G>T, p.E1011X, het	Exon 24	5´ of TM	ND
61	Female	**c.2345-2A>G, het**	Intron 18		*De novo*
62	Female	**c.2071T>A, p.C691S, het** and no mutation by MLPA	Exon 16	EGF	ND
63	Female	No mutation by sequencing and MLPA			
64	Male	No mutation by sequencing and MLPA			
65	Male	No mutation by sequencing and MLPA			
66	Male	**c.897delC, p.C300VfsX112, het**	Exon 7	EGF	ND
67	Male	**c.3194_3195delGA, p.R1065NfsX43, het**	Exon 25	5´ of TM	*De novo*
68	Male	**c.1281_1282insT, p.K428X, het**	Exon 10	EGF	*De novo*
69	Male	**c.2990C>A, p.S997X, het**	Exon 24	CR	Maternal
70	Female	**c.1382_1383delAC, p.D461GfsX8, het**	Exon 11	EGF	*De novo*
71	Male	**c.1931delG, p.C644SfsX99, het**	Exon 15	EGF	Paternal
72	Male	**c.2287_2288insAACG, p.G763EfsX24, het**	Exon 18	EGF	*De novo*
73	Female	**c.1885+3_1885+4insGT, het**	Intron 14		*De novo*
74	Male	**c.65delG, p.C22LfsX24, het**	Exon 1	5´ of DSL	ND
75	Female	**c.1118delC, p.T373KfsX39, het**	Exon 8	EGF	*De novo*
76	Male	c.2698C>T, p.R900X, het	Exon 23	CR	*De novo*
77	Male	No mutation by sequencing; MLPA not done			
78	Male	c.703C>T, p.R235X, het	Exon 5	5´ of EGF	ND
79	Female	No mutation by sequencing and MLPA			
80	Male	No mutation by sequencing; MLPA not done			
*81*	*Female*	*c*.*826delT*, *p*.*C276VfsX134*, *het*	Exon 6	EGF	ND
82	Male	c.439+1G>A, het	Intron 3		ND
83	Male	No mutation by sequencing and MLPA			
84	Female	No mutation by sequencing and MLPA			
85	Female	No mutation by sequencing and MLPA			
86	Male	No mutation by sequencing; MLPA not done			
87	Female	No mutation by sequencing and MLPA			
88	Male	c.3006C>A, p.C1002X, het	Exon 24	CR	ND
89	Male	No mutation by sequencing and MLPA			
90	Female	No mutation by sequencing and MLPA			
91	Male	No mutation by sequencing; MLPA not done			

Novel variants are in bold font; cases previously reported are in italic font. Conserved regions of JAG1 protein include the signal peptide (SP), the delta-serrate-lin12-like region (DSL), epidermal growth factor (EGF)-like repeats, the cysteine-rich (CR) region, and the transmembrane (TM) domain; 5´ of DSL: the region between SP and DSL domain; 5´ of TM: the region between CR and TM.

het: heterozygous; MPLA: multiplex-ligation-dependent probe amplification; ND: not done.

At the time of the study, seven synonymous and three missense variants were regarded as polymorphisms. Two of the missense variants were not seen in 1000 Genomes and single-nucleotide polymorphism (SNP) databases (Table 3); c.1511A>G (p.N504S) was identified in case 67 and inherited from his healthy mother, and c.3178C>T (p.R1060W) was identified in case 29 and inherited from his mildly affected mother. Both of these cases had simultaneous, definite disease-causing mutations. Therefore, c.1511A>G and c.3178C>T were considered as rare SNPs, rather than disease-causing mutations.

**Table 3:** *JAG1* polymorphisms identified in these cases.

Polymorphism	Amino acid	Location	dbSNP identifier	MAF (global)
c.267G>A	p.G89G	Exon 2	rs1051415	0.08
c.588C>T	p.C196C	Exon 4	rs1801138	0.16
c.765C>T	p.Y255Y	Exon 6	rs1131695	0.41
^a^c.1511A>G	p.N504S	Exon 6	-	-
c.2214A>C	p.T738T	Exon 17	rs1801140	0.09
c.2612C>G	p.P871R	Exon 22	rs35761929	0.04
c.3141G>A	p.S1047S	Exon 25	rs202075581	< 0.01
^**b**^ **c.3178C>T**	**p.R1060W**	Exon 25	-	-
c.3417T>C	p.Y1139Y	Exon 26	rs1051419	0.67
c.3528C>T	p.Y1176Y	Exon 26	rs1051421	0.21

^a^identified in case 67 and maternal;

^b^identified in case 29 and maternal;

Novel variant is in bold font.

dbSNP: single nucleotide polymorphism database; MAF: minor allelic frequency.

### Predicted effects of missense variants

*In silico* studies using two different functional prediction programs (Mutation Taster and Polyphen-2) predicted a deleterious impact from missense variants c.238A>G (p.K80E), c.550C>T (p.R184C), c.766G>T (p.G256C), c.1156G>A (p.G386R), c.2026T>G (p.C676G), c.2071T>A (p.C691S), c.2612C>G (p.P871R), and c.3178C>T (p.R1060W) (Table 4). Mutation c.1511A>G (p.N504S) was classified as disease-causing by Mutation Taster, but benign by Polyphen-2.

**Table 4:** Deduced effects of missense variants.

Missense variants	Mutation Taster		Polyphen-2	
	Prediction	*P* value	Prediction	*P* value
c.238A>G, p.K80E	Disease-causing	0.999	Probably damaging	0.997
*c*.*550C>T*, *p*.*R184C*	Disease-causing	0.999	Probably damaging	1.000
*c*.*766G>T*, *p*.*G256C*	Disease-causing	0.999	Probably damaging	1.000
*c*.*1156G>A*, *p*.*G386R*	Disease-causing	0.999	Probably damaging	0.997
c.1511A>G, p.N504S	Disease-causing	0.999	Benign	0.007
c.2026T>G, p.C676G	Disease-causing	0.999	Probably damaging	1.000
c.2071T>A, p.C691S	Disease-causing	0.999	Probably damaging	0.973
c.2612C>G, p.P871R	Disease-causing	0.999	Possibly damaging	0.703
c.3178C>T, p.R1060W	Disease-causing	0.999	Possibly damaging	0.586

Cases previously reported are in italic font.

### Distribution of JAG1 point and frameshift mutations

Mutations identified by sequencing occurred throughout the coding sequence of *JAG1*, and no common mutations were detected. The sequencing of eleven exons (exons 3, 5, 6, 11, 14, 16, 18, 21, and 23–25) of *JAG1* would detect a majority (69.2%) of the point and frameshift mutations.

### Segregation testing

A total of 37 sets of parents’ samples were available. Segregation testing revealed that 30/37 (81.1%) mutations arose *de novo*, whereas 7/37 (18.9%) were maternally (*n* = 4) or paternally (*n* = 3) inherited (Table 2).

### Mutation detection rate

Overall, a mutation in *JAG1* was identified in 70/91 (76.9%) cases. Fifty-five patients met the diagnostic criteria for ALGS; 20 patients with evidence of bile duct paucity had at least three clinical features of ALGS, and the remaining 35 patients had at least four clinical features. *JAG1* mutations were identified in 87.3% (48/55) of them (Table 1).

Thirty-six patients who did not meet the diagnostic criteria were considered as suspected ALGS cases. Although at least one examination was missing in 23 of these cases, 20 patients did not meet the clinical diagnostic criteria for definite ALGS (cases 56–65 and 78–87); *JAG1* mutations were detected in 50.0% (10/20) of them (Table 1).

## Discussion

In this study, *JAG1* mutations were identified in 87.3% of clinically diagnosed ALGS patients and 50% of clinically suspected ALGS patients, indicating that *JAG1* mutations are the major cause of classical ALGS, but also cause sporadic atypical Chinese ALGS cases. The mutation spectrum in this Chinese cohort included 45 novel mutations in *JAG1*, which is different from other populations [11–13]. The majority (69.2%) of the point and frameshift mutations would be detected with sequencing of only eleven of the *JAG1* exons (exons 3, 5, 6, 11, 14, 16, 18, 21, and 23–25), while 62% of mutations were detected in ten exons (exons 2, 4, 5, 6, 9, 12, 17, 18, 23 and 24) in other populations [13]. With the exception of six missense mutations, all other identified mutations (34 frameshift, 12 nonsense, 7 splicing, and 3 gross deletion) were predicted to result in a truncated protein.

According to the segregation testing, 81.1% of the mutations were *de novo*, which is higher than reported previously [6, 8, 11, 15]. Moreover, a frameshift mutation was identified in case 11, and his two brothers and one sister all died of suspected ALGS, though the sequencing of *JAG1* in his parents was normal, despite the fact that his mother had facial features characteristic of ALGS. We speculate that germline mosaicism occurred in this family, and sequencing only the DNA from blood samples failed to identify the mutation. Giannakudis *et al* [16] reported that the frequency of mosaicism for *JAG1* mutations in ALGS is > 8.2%, which should not be overlooked in genetic counseling.

In this cohort, nine missense variants were detected, including six mutations and three SNPs. Among these, c.550C>T, c.766G>T, and c.1156G>A were reported in our previous study [14]. The *de novo* mutations c.238A>G and c.2026T>G, as well as c.2071T>A, are novel and predicted to be deleterious; c.2026T>G and c.2071T>A are located in the conserved region of *JAG1*. As these three missense variants were not detected in the 1000 Genomes database and no other definite disease-causing mutations were identified, it is presumed that they are disease-causing mutations. Although the missense c.1511A>G has been reported as a disease-causing mutation [13], it was considered as a rare SNP in this study as it was also detected in the unaffected mother and occurred along with a *de novo* deletion (c.3194_3195delGA) that would result in a truncated protein product. Similarly, the novel missense c.3178C>T in case 29 was also considered as a rare SNP, as it occurred concurrently with a splicing mutation (c.755+2T>G) resulting in congenital heart disease that was inherited from his mother. Additionally, c.2612C>G (p.P871R) was detected with a concurrent nonsense mutation in case 59, with a global mean allelic frequency of 0.04 according to the SNP database. These findings provide evidence that segregation analysis is not only useful for genetic counseling, but also for judging the pathogenicity of missense variants.

The expression and penetrance of ALGS is variable, and genetic diagnosis can be useful for atypical patients. In this study, *JAG1* mutations were identified in patients with only two or three clinical features of ALGS, consistent with the report of Guegan *et al* [17], indicating that *JAG1* mutations can cause sporadic atypical ALGS, and thus gene testing should be conducted for patients who do not meet the diagnosis criteria of ALGS. The mutation detection rate for clinically definite ALGS patients in this study was 87.3%, which is lower than the 94% reported by Warthen *et al* [13]. However, it is not clear if the missense mutations detected in their study were disease causing, which could account for the mutation detection rate difference.

A limitation of the present study is that not all patients received identical clinical assessment, and samples were not obtained from all parents for segregation testing, which is inevitable in a retrospective study. Furthermore, the parents with *JAG1* mutations did not undergo full physical examinations.

In conclusion, the findings show that the vast majority of Chinese patients with clinical features of ALGS exhibit *JAG1* mutations. In addition, the mutation spectrum within this cohort is different from other populations. Finally, half of the patients presenting with just two or three clinical features of ALGS had *JAG1* mutations, indicating that *JAG1* testing will be useful for the diagnosis of atypical ALGS patients.
